# Monitoring and Occurrence of Heavy PAHs in Pomace Oil Supply Chain Using a Double-Step Solid-Phase Purification and HPLC-FLD Determination

**DOI:** 10.3390/foods11182737

**Published:** 2022-09-06

**Authors:** Laura Barp, Sabrina Moret, Giorgia Purcaro

**Affiliations:** 1 Department of Agri-Food, Environmental and Animal Sciences, University of Udine, 33100 Udine, Italy; 2 Gembloux Agro-Bio Tech, University of Liège, Chimie des Agro-Biosystèmes, Passage des Déportés 2, 5030 Gembloux, Belgium

**Keywords:** polycyclic aromatic hydrocarbons (PAHs), olive oil, pomace oil, solid-phase extraction (SPE), high performance liquid chromatography-fluorometric detector (HPLC-FLD)

## Abstract

Polycyclic aromatic hydrocarbons (PAHs) are ubiquitous environmental and processing contaminants generated by both spontaneous and anthropogenic incomplete combustion processes of organic matter. Contamination of PAHs in vegetable oils can result from several factors and processes, including environmental contamination, oil processing, and migration from food contact materials. The determination of PAHs in edible oil presents a challenge because of the complexity of the matrix. Since PAHs are present at lower levels than triglycerides, it is necessary to isolate the compounds of interest from the rest of the matrix. To this purpose, a new purification approach based on a double solid-phase extraction (SPE) step followed by high performance liquid chromatography–fluorometric detector (HPLC-FLD) analysis was developed. The method involves a first purification step by using a 5 g silica SPE cartridge, previously washed with dichloromethane (20 mL), dried completely, and then conditioned with *n*-hexane (20 mL). The triglycerides are retained by the silica, while the PAH-containing fraction is eluted with a mixture of *n*-hexane/dichloromethane (70/30, *v/v*). After evaporation, the residue is loaded on a 5 g amino SPE cartridge and eluted with *n*-hexane/toluene (70/30, *v/v*) before HPLC-FLD analysis. The focus was the evaluation of the contribution of the various phases of the pomace oil supply chain in terms of the heavy PAHs (PAH8) concentration. Data collected showed that pomace contamination increased (by 15 times) as storage time increased. In addition, the process of pomace drying, which is necessary to reduce its moisture content before solvent extraction of the residual oil, appeared to significantly contribute to the total heavy PAHs content, with increases in value by up to 75 times.

## 1. Introduction

Polycyclic aromatic hydrocarbons (PAHs) are a large class of compounds characterized by the fusion of two or more aromatic rings and are classified into light and heavy according to the number of benzene rings, i.e., 2–3 in the former case and 4–6 in the latter [1,2,3,4]. Due to their chemical structure, PAHs are poorly soluble in water but highly affinitive to non-polar solvents and oils [4,5,6,7]. PAHs are ubiquitous environmental and processing contaminants generated by both spontaneous and anthropogenic incomplete combustion processes of organic matter [2,3,6]. PAHs can affect human health through various harmful effects, mostly related to carcinogenesis and mutagenesis in addition to immunosuppressive effects [1,2,3]. Humans are exposed to PAHs through a variety of routes (ingestion, inhalation, skin contact), the primary route being food [8,9]. Among the different food categories, oil, and fats are the major contributors of PAHs to the human diet, either directly or indirectly through their incorporation into formulations such as cookies and cakes [10].

Contamination of PAHs in vegetable oils can result from several factors and processes, including environmental contamination, oil processing, and migration from food contact materials [4]. Contamination of virgin olive oil can occur either directly during the processing in the mill or indirectly due to olive superficial contamination via atmospheric fallout [2]. Once the oil is mechanically extracted from the olives to produce the virgin oil, the olive residue, called olive pomace, still contains roughly 6–7% oil [11]. Since no more profitable methods of using pomace have been identified at present, it is considered economically viable to proceed with oil recovery through a solvent extraction. Wet pomace, just after oil extraction, is stored (first in the mill and then in the pomace factory) for a relatively long period prior to being dried via direct contact with combustion fumes, often produced by burning the spent pomace, significantly contributing to PAHs contamination. The dried solid mass (5–8% residual moisture) is then extracted with hexane, and the resulting solution is distilled to eliminate the solvent and recover the oil [4,9,11]. In turn, the spent pomace can be used as fuel or, after separation of the woody part, as livestock feed [12]. The crude pomace oil obtained by this process cannot be used directly for human consumption but requires a refining step to remove unwanted minor components, such as free fatty acids, color pigments, phospholipids, metals, and waxes [13]. At the end of all these processes, virgin oils are added to refined pomace oils to restore both the properties of resistance to autoxidation and the organoleptic characteristics lost in refining. Refining steps, particularly neutralization, are effective in reducing the PAHs content of olive pomace oil [14], however, considerable levels may remain due to the high initial content [4].

From a legislative point of view, PAHs in foodstuffs are strictly regulated by the Commission Regulation (EU) n. 835/2011, which defines a limit of 10 μg/kg for the sum of four PAHs (PAH4: benzo(a)pyrene—BaP, chrysene—Ch, benzo(a)anthracene—BaA, benzo(b)fluoranthene—BbF) and 2 μg/kg for BaP in vegetable oils [15]. Moreover, Regulation (EU) 836/2011 defines the analytical performances required by the method for analyzing them [16]. 

Over the years, many analytical methods have been proposed for PAHs analysis in different food matrices; for more information, the readers are directed toward the many reviews available in the literature [3,6,8,9,17,18,19,20]. Herein, we will focus on the determination of PAHs in edible oil, which is particularly challenging due to the high affinity of the PAHs with the whole composition of the matrix [1,4]. Since PAHs are present at lower levels than triglycerides, it is necessary to isolate the compounds of interest from the rest of the matrix [1,21]. For this purpose, several methods of analysis of PAHs in fats and oils have been developed, involving an extraction and purification step before the analytical determination by using liquid (LC) or gas chromatography (GC) [1,4,21,22,23,24,25]. Among the pretreatment methods commonly used (such liquid–liquid extraction, saponification, etc.) solid-phase extraction (SPE) allows the combination of the extraction and purification steps, with the direct application of the sample on the cartridge, with or without dilution in an appropriate solvent [1,4,21,26,27,28,29,30,31,32,33,34,35]. Different stationary phases, such as silica (C18), Florisil, alumina, styrene-divinylbenzene, and different elution solvents, such as hexane, dichloromethane, cyclohexane, and tetrahydrofuran, have been employed [30,31,32,33,34,35]. After properly obtaining the extract, elution solvents are generally evaporated, and the residue is usually redissolved in an appropriate solvent prior to the injection into the chromatographic instrument. Concerning analytical determination, gas chromatography–mass spectrometry (GC-MS) has been widely used for the analysis of PAHs [21,23,31,36,37,38,39], but the selection of an appropriate stationary phase for the separation of PAHs isomers and the high discrimination observed for high molecular weight compounds have to be overcome [40]. Tandem MS (MS-MS) coupled to GC has been proposed to overcome deficiencies in the quantification of heavier PAHs that tend to co-elute with other similar compounds [41,42] as well as multidimensional analytical techniques, such as comprehensive GC (GC × GC), to chromatographically separate the isomers before detections [43,44]. LC coupled to a ultraviolet/visible (UV-VIS) diode-array detector (DAD) was also used for the determination of PAHs. However, a florescence detector (FLD) is considered the best option because of its superior specificity and sensitivity [27]. LC-MS has limited use in the determination of PAHs in oils, most likely because of the difficulty of obtaining efficient ionization [8,45,46].

The aim of the present work is to evaluate the effect of the various production phases, starting from olives and ending with crude olive pomace oil, in terms of the increase in heavy PAHs concentration, which to the authors’ knowledge has never been investigated before. The focus was on the PAHs requested to be monitored by the European Food Safety Authority (EFSA) in 2008 [47]: a set of eight PAHs (PAH8: BaA, Ch, BbF, BaP, benzo(k)fluoranthene—BkF, dibenzo(a,h)anthracene—DBahA, benzo(g,h,i)perylene—BghiP, indeno(1,2,3-cd)pyrene (IP) and a subgroup of four PAHs (PAH4: BaP, Ch, BaA, BbF). The latter were then regulated in the EU Reg. 835/2011 [15]. To overcome difficulties in the analysis due to the complexity of the pomace oil matrix and the presence of numerous interfering compounds, a purification step based on a double SPE step followed by high performance liquid chromatography (HPLC) FLD analysis was applied.

## 2. Materials and Methods

### 2.1. Reagents and Standards

Acetonitrile, dichloromethane, *n*-hexane, and toluene, all of HPLC grade, were purchased from Merck (Darmstadt, Germany). Deionized water was obtained by using a Milli-Q system (Millipore, Bedford, MA, USA). Silica cartridges for SPE (5 g, 20 mL and 2 g, 12 mL, Mega Bond Elut) and amino cartridges (5 g, 20 mL) were from Varian (Palo Alto, CA, USA). The standard PAHs mixture, 610 M, in 1 mL of methanol/dichloromethane (Supelco, Bellefonte, PA, USA) consisted of: acenaphthene (1000 μg/mL, 99.0%), fluoranthene (200 μg/mL, 99.0%), naphthalene (1000 μg/mL, 96.9%), BaA (100 μg/mL, 99.0%), BbF (200 μg/mL, 99.1%), BaP (100 μg/mL, 99.5%), BkF (100 μg/mL, 99.6%), Ch (100 μg/mL, 99.0%), acenaphthylene (2000 μg/mL, 99.0%), anthracene (100 μg/mL, 99.0%), BghiP (200 μg/mL, 99.0%), fluorene (200 μg/mL), phenanthrene (100 μg/mL, 97.2%), DBahA (200 μg/mL, 96.7%), IP (100 μg/mL, 99.7%), and pyrene (100 μg/mL, 97.4%).

### 2.2. Samples

Samples of olives (4), virgin olive oils (6), olive paste (1), and corresponding fresh pomace (5) were collected from different mills. Samples of pomace (25) were collected at different times of storage from both olive mills and pomace factories. About 1 kg of each product was sampled, homogenized, prepared, and analyzed as described below. 

### 2.3. Oil Extraction

The olive samples were coarsely crushed with the help of a hammer. A representative aliquot of the solid samples (olives, olive paste, residual pomace) was then dried in an oven at 75 °C for 12 h to remove moisture and thus facilitate solvent extraction. Next, the samples were ground, and a 15 g aliquot was added to 40 mL of hexane and subjected to ultrasound-assisted extraction for 1 h in the case of olive samples and 2 h in the case of pomace samples. The extract was then filtered on paper (Whatman™ Grade 1), collected in a flask along with the washes (5 × 3 mL of hexane), and, finally, the solvent was removed from the extracted oil by means of a rotary evaporator until a constant weight was achieved. 

### 2.4. Sample Purification

**Figure 1:**
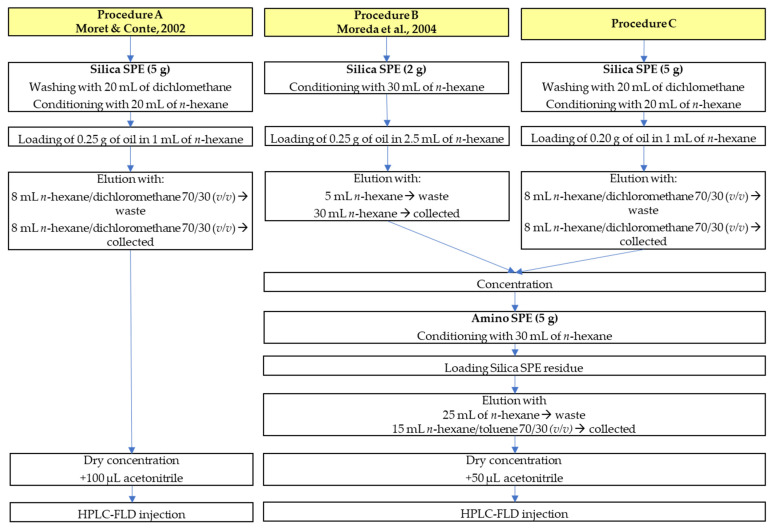
Flow chart of SPE protocol comparison for olive pomace oil purification. Moret & Conte, 2002 [34]; Moreda et al., 2004 [35].

The different tested approaches to sample purification are schematized in the flowchart shown in Figure 1. The starting methods where those proposed by Moret and Conte (2002) [34] and Moreda et al. (2004) [35]. The first approach involves a first purification step using a 5 g silica SPE cartridge previously washed with dichloromethane (20 mL), dried completely, and then conditioned with *n*-hexane (20 mL). The triglycerides are retained by the silica, while the PAH-containing fraction is eluted with a mixture of *n*-hexane/dichloromethane (70/30, *v/v*) (Figure 1—Procedure A). The PAH-containing fraction was concentrated under a nitrogen flow, and the residue dissolved in acetonitrile was directly injected (20 μL) into the HPLC-FLD system. This method was used for all the oil samples obtained from olives and fresh pomace.

The method proposed by Moreda et al. [35] involves a first purification step using a 2 g silica SPE cartridge conditioned with *n*-hexane (30 mL), and the PAH fraction is eluted with 30 mL of *n*-hexane. After evaporation, the residue is loaded on a 5 g amino SPE cartridge before HPLC-FLD analysis (Figure 1—Procedure B). The combination of methods A and B resulted in a new procedure (Figure 1—Procedure C), which involves a first purification step using a 5 g silica SPE cartridge and a second one using a 5 g amino SPE cartridge. This new approach was used to analyze heavy PAHs in crude pomace oil and oil extracted from dried spent pomace.

### 2.5. HPLC-FLD Analysis

The analytical determination of PAHs was carried out with a Varian model 9010 HPLC gradient pump equipped with a Rheodyne 7161 injector with a 30 μL loop. The column was a C18 reversed phase (250 mm × 3 mm, i.d. 5 μm, Supelcosil LC-PAH, Supelco) thermostatted at 38 °C with a column heater (model L 7350, LaChrom, Merck, Darmstadt, Germany). The mobile phase consisted of acetonitrile and water at a flow rate of 1 mL/min. The gradient elution program started with 40% acetonitrile (isocratic for 5 min), reaching 100% of acetonitrile in 40 min. PAHs detection was carried out with a Jasco spectrofluorometer (model FP 1520, Cremalla, Como, Italy) using two different wavelength sequences, reported in Table 1.

The absence of the matrix effect was assessed previously by Purcaro et al. [48]. 

The peaks of heavy PAHs in the samples were identified by comparison of retention times with those for a standard mixture and manually integrated. For quantitative calculations, data were corrected on the basis of the blank analysis, thus avoiding a non-negligible source of error. An external calibration curve has been used.

### 2.6. Method Performances

Validation of the procedure was carried out by spiking a crude pomace oil sample, analyzed in triplicate (*n* = 3) by using the method described in Figure 1—Procedure C. Method performance criteria are reported in Table 2: mean recovery (%), standard deviation (SD), relative standard deviation (RSD%), the limit of detection (LOD = μ_b_ + 3 σ_b_), the limit of quantification (LOQ = μ_b_ + 10 σ_b_), the linearity range (to stay in range, samples with high PAHs contents were diluted), and the Horwitz Ratio (HorRat, calculated by applying the modified Horwitz equations for concentrations lower than 120 μg/kg). All the performance criteria required by the Reg. 836/2011 for the analysis of BaP, BaA, BbF, and Ch in foods were verified. 

## 3. Results and Discussion

### 3.1. Method Optimization 

**Figure 2:**
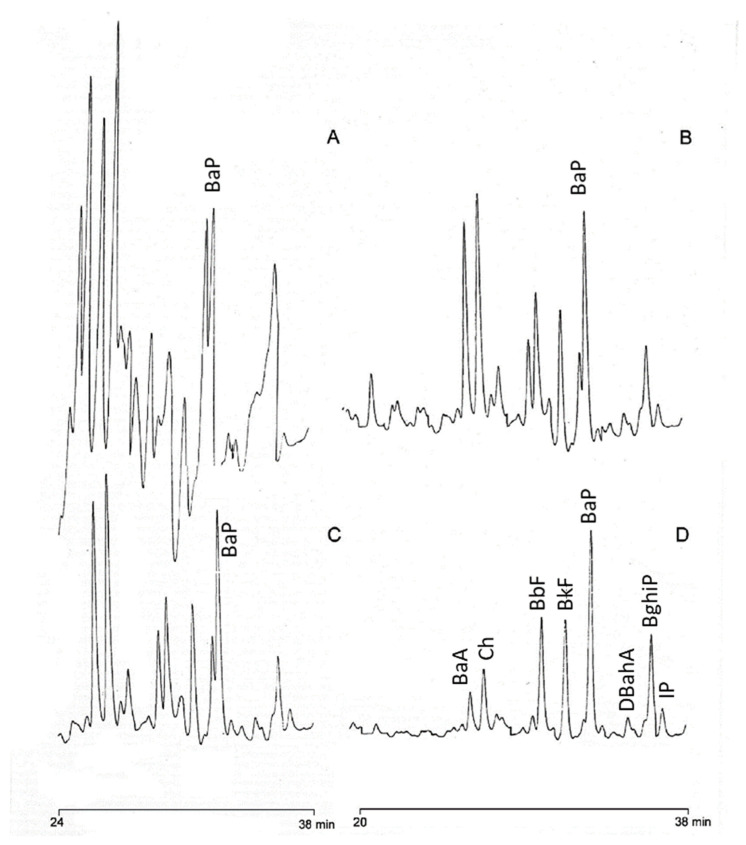
HPLC-FLD chromatograms of the same sample of crude pomace oil purified by following procedure proposed by (**A**) Moret and Conte (2002) [34], (**B**) by Moreda et al. (2004) [35], (**C**) by using the new proposed approach, and (**D**) by using the new proposed approach but reducing the elution fraction.

While the method previously developed by Moret and Conte (2002) [34] (Figure 1—Procedure A) could be used for the determination of oil extracted from olives, for oils obtained from the extraction of spent pomace and crude pomace oil, the use of this method resulted in an interferent-rich chromatogram (Figure 2A). In particular, in the heavy PAHs elution zone, there are numerous interferent compounds (probably methyl and ethyl PAHs derivatives) evidently not separated in the sample purification step and making a proper quantification of the analytes of interest difficult. In an attempt to improve the reliability of the results, the method proposed by Moreda et al. [35] (Figure 1—Procedure B) was tested. This approach involves a 2 g silica SPE cartridge to isolate PAH-containing fraction and a further purification step on a 5 g amino SPE cartridge, from which the fraction of interest was desorbed by using a mixture of *n*-hexane/toluene. The HPLC-FLD chromatogram obtained analyzing a sample of crude olive pomace oil previously purified by applying this method was significantly improved in terms of the degree of purification of the analytes of interest, although interfering peaks are still present (Figure 2B).

Combining the two methods previously described [34,35], that is, using a first 5 g silica SPE cartridge and a second 5 g amino SPE cartridge, similar results were obtained in terms of chromatogram cleanliness (Figure 2C) but with the advantage of reducing the amount of solvent used for eluting the compounds of interest (Figure 1—Procedure C). In particular, by using more silica phase (5 g instead of 2 g), which ensures adequate retention of triglycerides, and increasing the polarity of the elution solvent (mixture of *n*-hexane/dichloromethane instead of *n*-hexane alone), it was possible to collect the fraction of interest in only 8 mL instead of 30 mL, thus also shortening the evaporation time.

**Figure 3:**
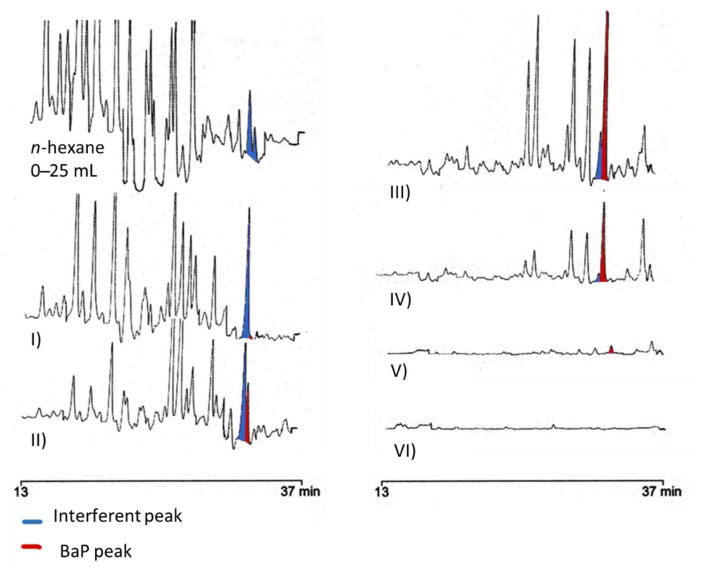
HPLC-FLD traces obtained by fractionation of a crude pomace oil sample on a 5 g amino SPE cartridge. Subsequent fractions, consisting of 3 mL of *n*-hexane/toluene (70/30, *v/v*) are marked by Roman numerals (from I to VI).

In order to evaluate the possibility of further improving sample purification in the amino SPE, the fractionation process was investigated in more detail. A sample of crude pomace oil, previously purified on silica (5 g), was fractionated on the 5 g amino SPE cartridge, collecting successive fractions of 3 mL each during the elution with the *n*-hexane/toluene 70/30 (*v/v*) mixture. The obtained fractions were then concentrated and injected into the HPLC-FLD system (Figure 3).

In the first discarded fraction (0–25 mL of *n*-hexane) numerous interferents were present in the elution zone of heavy PAHs—in particular, BbF, BkF, and BaP. The next two fractions (I: 0–3 and II: 3–6 mL), eluted with the *n*-hexane/toluene mixture (70/30, *v/v*), still showed the presence of interferents, particularly a peak that elutes just before BaP (highlighted in blue in Figure 3). Only from fraction III (6–9 mL), the BaP peak becomes prevalent on the interferent compound. An attempt to obtain a cleaner chromatogram by removing most of the interfering compounds was made by also removing the first 6 mL eluted with hexane/toluene (fractions I and II in Figure 3), thereby narrowing the fraction collected (fractions III, IV and V in Figure 3: from 6 to 15 mL eluted with *n*-hexane/toluene 70/30, *v/v*). Although this procedure resulted in a particularly interference-free HPLC-FLD chromatogram (Figure 2D), there was a drastic decrease in PAHs recoveries—in particular, BaA, with a recovery of 10.4% (*n* = 3, RSD%: 25.2)—making this route unfeasible. The new proposed purification approach (Figure 1—Procedure C) was tested in terms of recoveries, evaluated (*n* = 3) on a crude pomace oil sample fortified with a known amount of PAHs. The same spiked sample was also purified by using the procedure reported in Ref. [35] (Figure 1—Procedure B), previously applied to a strongly refined olive pomace oil sample. Recoveries higher than 79% were obtained for all heavy PAHs. Mean percentage recoveries, standard deviations, and relative standard deviations (RSD%) obtained are reported in Table 2.

Recoveries obtained by using Procedure B [35] were all around 100%, with the exception of BaA, the first eluted heavy PAH, which had a recovery of about 71%. However, SD and RSD% were higher than 20 and 30, respectively. Using the C-sample purification procedure, the lowest recovery was BaA at about 60%, followed by Ch with 78%. Compared with Procedure B, there are better SD and RSD% values of under 20 and 25, respectively. In addition, there is a slight increase in the cleanliness of the chromatographic trace (Figure 2), which made the authors lean toward choosing procedure C as a good compromise between decreasing interferents, throughput, and a satisfactory quantitative analysis. Procedure C was ultimately validated by calculating the LOD, LOQ, and HorRat values, as requested by the European Reg 836/2011. All the values satisfied the requested performances.

### 3.2. Heavy PAHs along the Olive and Pomace Oil Supply Chain

Virgin olive oil is extracted from the olive fruit exclusively by mechanical processes without any further treatment. Generally, the process comprises a series of steps including olive harvesting (manually or mechanically), transportation to olive mills, washing, crushing, mixing the olive paste, and oil separation via centrifugation or pressing. One of the main sources of contamination in this case is represented by environmental pollution—in particular, dust and particles from smoke and air pollution that may contaminate olive skin.

Table 3 reports the content of PAHs found during the olives’ transformation into virgin oil and on the residual pomace and the following steps of pomace transformation to pomace oil. The data reported in Table 3 showed that olives of different variety collected in different olive mills (OM) had low contamination levels of heavy PAHs, generally below 5 μg/kg. The same applies to the virgin olive oil samples analyzed (PAH8 below 4 μg/kg). Comparing the results obtained by extracting oil from the same olive sample both using solvent in the laboratory and via centrifugation (4.3 and 1.4 μg/kg, respectively) clearly showed the higher extraction of total heavy PAHs obtained by the chemical method. The extraction of PAHs with oil is never quantitative; therefore, a residual contamination was found even in olive paste. These heavy PAHs levels are related both to the starting raw material as well as to the reconcentration effect of the pollutants due to the fact that they are extracted in less oil. In all samples the legal limit of 2 μg/kg for BaP was never exceeded, while the limit of 10 μg/kg for PAH4 (BaA, Ch, BbF, BaP) was exceeded in two samples, both of residual pomace oil. The largest contribution to the sum PAH4 and PAH8 comes from Ch, which reached considerable levels (up to 7.9 μg/kg) in particular in residual pomace oil samples. After the oil is extracted by physical means from the olives, the residual pomace goes through long periods of storage during which it may be subject, depending also on storage condition, to various possibilities of contamination, such as environmental and residues of mineral oils (i.e., motor and hydraulic oils) containing a certain amount of PAHs [4]. The pomace at the mill is transported by a conveyor belt outside the plant, usually to an open yard, where the pile formed is periodically moved with old and worn-out bulldozers (which may leak motor or hydraulic oil), to add the newly produced pomace (Figure S1). In addition, the pomace is also exposed to exhaust fumes from bulldozers or other vehicles driving in the yard, which is sometimes also used as a parking area, as well as to fallout from atmospheric particulate matter [4,27]. Moreover, a contribution due to contamination of lubricating oils used in extraction plants, handling lines, and asphalt dust residues during the transportation phase cannot be ruled out. The intrinsic characteristic of the exhausted pomace exacerbates the concentration factor of the PAHs. In fact, the lower the oil content, the greater the reconcentration effect of any environmental contaminants of a lipophilic nature: olive pomace contains an average of 3–5% residual oil on a dry basis, while whole olives have an average content of about 30% (thus a concentration effect of 6–10 times) [45].

Once the pomace arrives at the pomace mill, it is again stored outdoors for varying lengths of time depending on processing requirements, thus being subjected to the aforementioned sources of contamination in the olive mill. Finally, before solvent extraction, pomace must necessarily be dried. This process is often carried out using systems of direct contact of the combustion fumes with the fresh pomace mass, and often, the exhausted pomace itself is used as fuel, leading to a significant increase in the concentration of PAHs [45,46].

The dried pomace and crude pomace oil samples were analyzed using the new purification approach procedure proposed in this work (Figure 1—Procedure C). The focus was on heavy PAHs (PAH8 and PAH4), and data were corrected for recoveries (Table 2). Samples of pomace were taken at both olive mills (OM) and pomace factories (PF) (Table 4); storage times attributed to pomace are approximate and in accordance with the information provided in site during the sampling. 

The data reported in Table 4 showed that the storage of pomace, generally accumulated in yards (often used also as parking lots) outside both olive mills and pomace factories, contributed significantly to the total PAHs content as well as the pomace’s handling. Samples stored for several days presented significantly higher contamination levels with respect to the pomace samples processed in short periods. This is evident by comparing PAH8 levels of samples collected in OM1 and homogenized to obtain a representative sample of the whole mass: in particular, fresh pomace sample had a PAH8 content of 8.6 μg/kg, while the contamination level after 10–20 days of storage in the olive mill yard increased about 15 times, reaching 125.8 μg/kg. Larger increases were observed in the case of prolonged storage periods in pomace factories: fresh pomace samples collected in PF2 presented a total content of heavy PAHs of 4.4 μg/kg after 2–3 days storage and of 657.3 μg/kg after 15–20 days storage. In this case, the pomace was stored in the yard in front of the plant and was invested by fumes escaping from the dryer chimney. It was also handled with a bulldozer, which may have contributed to the contamination through leaking engine oil or hydraulic oil. 

Different contamination levels were also found depending on the depth of sampling in the same pomace pile. Samples collected in OM3 taken 20–30 cm deep in the pomace pile showed 4.3 μg/kg of PAH8 compared to 13.7 μg/kg of pomace taken superficially from the same pile. Furthermore, the 30-day pomace sample taken at the same oil mill but which was located in a more sheltered area of the yard showed low levels of heavy PAHs (4.5 μg/kg), comparable to those of the deep-sampled pomace. The same behavior was observed in samples stored for 7 days in PF2, where PAH8 levels were about 10 μg/kg on the surface and of 6 μg/kg in the depths of the same pomace pile. This highlights the importance of environmental contamination by atmospheric particulate matter and coming from exhausts of motor vehicles driving in the area where pomace stays. The results obtained for the pomace sample named “OM4—7–10 days” corroborated the previous consideration. In fact, this sample showed PAH8 levels (9.7 μg/kg) comparable to those of 2–3 days pomace samples (8.4 μg/kg) collected in the same olive mill. In this case, the pomace was stored in a masonry room with only one side open to a secondary road with little traffic. In addition, this sample was not subjected to bulldozer passes to compress the mass. The high PAHs contents in the two fresh pomace samples named OM6 and OM7 were related to the higher contents of contamination already found at the level of olives in this area and to the concentration effect (6–10 fold) that generally occurs in all pomace samples. 

The obtained data (Table 4) confirmed that pomace drying, generally conducted in plants that involve direct contact of the raw material with combustion fumes, was responsible for contamination with significant amounts of PAHs. This effect was clear in the “pomace entering the drying plant” sample from the PF2, which contained 0.8 μg/kg of BaP and a total of 4.1 μg/kg of heavy PAHs, while in the “dry pomace” sample (after the drying process), the BaP content increased to 34.1 μg/kg and that of PAH8 to 309.1 μg/kg, which is approximately 75 times more. According to indications provided in the pomace factory at the sampling time, they were processing a fresh batch of pomace which had just been delivered, which would explain the low levels of contamination found in the pomace prior to drying. 

Finally, data on the “oil/hexane mixture” and “crude oil” samples taken in both PF1 and PF2 seemed to indicate a contribution to pollution from the extraction phase, during which values in the order of mg/kg of heavy PAHs are reached. In particular, the “oil/hexane mixture” sample from PF1 had almost 8 mg/kg of PAH8, and the “crude oil” sample reached about 17.5 mg/kg; while the homonymous samples from PF2 had 1 mg/kg and almost 3 mg/kg of total heavy PAHs, respectively. 

Furthermore, the analysis of “hexane pre-extraction” and “hexane post-extraction” samples showed low levels of each PAH below the quantification limit in the first sample, while slightly higher PAHs values (ranging from 0.1 to 0.6 μg/kg) were found in the second. These results seemed to highlight how possible solvent contamination may be totally transferred to the oil during the extraction phase due to the contact with the hexane. However, since the hexane/oil ratio is unknown, it was not possible to calculate the solvent contribution, which is in any case too small to justify the particularly high levels found in the “oil/hexane mixture” and in the “crude oil” samples. In addition, hexane after the first extraction is distilled away from the oil, a practice that contributes to the cleanliness of the solvent, which then should not contribute to further contaminating the oil in subsequent extractions. High levels of PAH8 contamination (around 2 mg/kg) were also found in the “exhausted pomace” sample collected in a pomace factory in Puglia, which, as mentioned above, will then be used as fuel for drying other fresh pomace from the mills, promoting continuous and unending contamination.

Among samples collected at olive mills, the legal limit of 2 μg/kg for BaP was exceeded in three samples of pomace (OM2, 10–20 days pomace, 5.5 μg/kg; OM6, fresh pomace, 4.2 μg/kg; OM7, fresh pomace, 6.1 μg/kg). In contrast, the same legal limit was exceeded in more than half of the samples collected in pomace factories, reaching in some cases even values in the order of mg/kg.

## 4. Conclusions

The present work aimed to investigate the various stages of the pomace oil supply chain to understand the contribution of each to the total heavy PAHs contamination. For this purpose, a two-SPE-cartridge purification/separation approach was applied in order to eliminate most of the interfering compounds resulting from the complexity of the matrix and thus to obtain a better plot and reliable quantification of heavy PAHs. In particular, sample extracts were processed by using a first silica (5 g) SPE cartridge and a second amino phase cartridge (5 g) before the HPLC-FLD analytical determination. 

Data collected through the oil production chain showed that olives and olive oils had low contamination levels of heavy PAHs, mainly due to environmental pollution. A residual contamination was found in olive paste after oil extraction, related both to the starting raw material as well as to the reconcentration effect of the pollutants. Furthermore, pomace contamination increased (by 15 times) as storage time increased both in olive mills and in pomace factories. During this storage time, handling of the pomace with bulldozers can also contribute to the final contamination of PAHs due to possible leakage of engine oil or hydraulic oil. In addition, the process of pomace drying, which is necessary to reduce its moisture content before solvent extraction of the residual oil and generally conducted in direct contact with combustion fumes, appeared to significantly contribute to the total heavy PAH content, with increases in values by up to 75 times.

## Figures and Tables

**Table 1:** FLD wavelength program. λ_ex_: excitation wavelengths, λ_em_: emission wavelengths.

PAH	Time (min)	λ_ex_ (nm)	λ_em_ (nm)
BaA, Ch	23.0	270	390
BbF	28.0	260	430
BkF, BaP	30.7	256	410
DbahA, BghiP	34.0	290	410
IP	36.4	290	484

**Table 2:** Mean percentage recoveries, standard deviations (SD), and relative standard deviations (RSD%) obtained by applying the new proposed purification approach (Procedure C) and the method proposed by Moreda et al. [35] (Procedure B) on a spiked crude pomace oil sample (*n* = 3).

		Procedure B			Procedure C						
PAH	Spike (μg/kg)	Recovery (%)	SD	RSD%	Recovery (%)	SD	RSD%	LOD (μg/kg)	LOQ (μg/kg)	Linearity Range	HorRat
BaA	1.0	71.1	22.4	31.5	56.5	14.1	24.9	0.03	0.1	0.1–59.9	1.13
Ch	1.0	100.1	33.0	32.9	78.1	18.3	23.4	0.21	0.7	0.7–59.5	1.06
BbF	2.0	114.7	4.6	4.0	109.4	5.5	5.0	0.06	0.2	0.2–89.8	0.23
BkF	1.0	108.6	5.5	5.1	101.6	5.3	5.2	0.03	0.1	0.1–59.6	0.24
BaP	1.0	104.0	4.9	4.7	98.8	4.6	4.7	0.03	0.1	0.1–59.5	0.21
DBahA	2.0	101.5	1.2	1.2	96.3	1.6	1.6	0.03	0.1	0.1–89.1	0.07
BghiP	2.0	119.6	9.7	8.1	102.7	7.0	6.8	0.09	0.3	0.3–89.2	0.31
IP	1.0	115.6	5.7	4.9	94.2	10.7	11.4	0.03	0.1	0.1–59.5	0.52

**Table 3:** PAHs content (μg/kg) of samples collected in different olive mills.

Olive Mill	Sample Type	Extracted Oil (%)	BaA	Ch	BbF	BkF	BaP	DBahA	BghiP	IP	PAH4	PAH8
OM1	Picholine olives	26.6	0.2	2.7	0.1	<0.1	0.1	<0.1	0.2	0.1	3.1	3.4
	Olive paste	33.4	0.3	2.4	0.1	0.2	0.1	0.1	0.3	0.3	2.9	3.8
	Virgin olive oil		<0.1	1.3	0.1	<0.1	0.1	<0.1	0.2	0.2	1.5	1.9
	Residual pomace oil	8.1	1.8	7.9	1.9	0.9	0.5	<0.1	<0.3	0.5	12.1	13.5
OM2	Ghiacciola olives	19.7	<0.1	<0.7	0.1	<0.1	0.1	0.1	0.1	0.2	0.2	0.6
	Virgin olive oil		<0.1	<0.7	<0.2	<0.1	<0.1	<0.1	<0.3	0.1	-	0.1
	Residual pomace oil	4.3	0.6	3.3	2.1	0.7	0.5	0.3	0.4	0.7	6.5	8.6
OM3	Bianchera olives	31.5 (solvent)	0.4	3.1	0.4	<0.1	0.1	<0.1	0.1	0.2	4	4.3
	Bianchera olives	(centrifugation)	<0.1	0.9	0.1	<0.1	0.1	0.1	<0.3	0.2	1.1	1.4
	Virgin olive oil-01 *		0.1	2.9	0.1	0.1	0.1	<0.1	0.2	0.2	3.2	3.7
	Virgin olive oil-02 **		<0.1	1	<0.2	<0.1	0.1	<0.1	0.4	0.1	1.1	0.7
	Residual pomace oil	7.2	1.5	6.2	2.2	0.7	0.1	0.1	0.3	0.6	10	11.7
OM4	Virgin olive oil		<0.1	0.2	<0.2	<0.1	0.1	<0.1	<0.3	0.1	0.3	0.4
	Residual pomace oil	3.5	0.9	5	0.8	0.4	0.5	<0.1	0.3	0.5	7.2	8.4
OM5	Virgin olive oil		<0.1	0.4	<0.2	<0.1	0.1	<0.1	0.1	0.2	0.5	0.8
	Residual pomace oil ***	16	0.2	1.4	0.1	0.2	0.3	<0.1	0.2	0.3	2	2.7

* oil extracted with a centrifugal system. ** oil extracted from the same olives using a mini pressure plant. *** pomace from Sinolea plant.

**Table 4:** Heavy PAHs content, expressed as μg/kg, in pomace samples collected in different olive mills (OM) and pomace factories (PF).

OM/PF	Sample Type	Extracted Oil %	BaA	Ch	BbF	BkF	BaP	DBahA	BghiP	IP	PAH4	PAH8
OM1	fresh pomace	8.1	1.8	7.9	1.9	0.9	0.5	<0.1	<0.3	0.5	12.1	13.5
OM2	fresh pomace	4.3	0.6	3.3	2.1	0.7	0.5	0.3	0.4	0.7	6.5	8.6
	1–2 days pomace	4.6	0.9	4.1	0.7	0.4	0.4	0.1	0.5	0.3	6.1	7.4
	10–20 days pomace	5.7	71.4	26.4	11.3	4.9	5.5	0.5	3.1	2.7	114.6	125.8
OM3	fresh pomace	14.9	1.5	6.2	2.2	0.7	0.5	0.1	0.3	0.6	10.4	12.1
	10–20 days pomace—surface	5	2.8	<0.7	4.5	1.6	1.8	0.2	1.5	1.3	9.1	13.7
	10–20 days pomace—depth	4.4	0.7	1.6	0.4	0.3	0.5	<0.1	0.4	0.4	3.2	4.3
	30 days pomace—surface—clean area	2.6	0.1	0.5	1.9	0.1	0.6	0.2	0.4	0.7	3.1	4.5
OM4	2–3 days pomace	3.5	0.9	5,0	0.8	0.4	0.5	<0.1	0.3	0.5	7.2	8.4
	7–10 days pomace	4.3	1.1	5.8	0.9	0.5	0.5	0.4	0.2	0.3	8.3	9.7
OM5	fresh pomace—decanter		0.5	1.7	0.2	0.3	0.3	0.4	0.2	0.5	2.7	4.1
	fresh pomace—sinolea	16	0.2	1.4	0.1	0.2	0.3	<0.1	0.2	0.3	2,0	2.7
OM6	fresh pomace	3.8	3,0	11.6	0.2	4.8	4.2	0.7	4.5	2.1	18.8	30.9
OM7	fresh pomace	5.9	5.2	13.7	1.7	4.3	6.1	0.3	2.8	4.1	26.7	38.2
PF1	fresh pomace freshly conferred	3.9	1.1	5.9	1.4	0.7	1.5	<0.1	1.1	1.1	9.9	12.8
	fresh pomace conferred by 15 days	2.5	95.5	44.5	30.2	11.9	24.2	1.1	21.1	22.2	194.4	250.7
	dry pomace	5.6	319	901.5	197.7	69.1	58.2	15	97.2	152.5	1476.4	1810.2
	oil/hexane mixture		1052.7	1533	1481.9	462.3	1064.1	97.2	1002.8	1271.4	5131.7	7965.4
	crude oil		2816.5	3613.2	2888.6	886	3081.2	190.5	1955.7	2127.2	12399.5	17558.9
	oil from fresh exhausted pomace	1.3	357	477.1	753.3	238.3	819.6	96	1237.5	1345.4	2407	5324.2
PF2	fresh pomace 7 days—surface	6.9	0.3	5.9	1	0.4	0.8	0.1	0.9	0.8	8	10.2
	fresh pomace 7 days—depth	5.4	0.1	3.8	0.8	<0.1	0.5	0.1	0.3	0.6	5.2	6.2
	fresh pomace 7 days—yard contact	6.1	0.3	4.1	0.6	<0.1	0.9	0.1	0.8	0.5	5.9	7.3
	fresh pomace 2–3 days	3.3	0.1	2.8	0.6	<0.1	0.4	<0.1	0.1	0.4	3.9	4.4
	fresh pomace 15–20 days	3.6	126.7	219.8	104.8	46.2	67.6	4	43.1	45.1	518.9	657.3
	fresh pomace entering the drying plant	4.5	<0.1	2	0.5	<0.1	0.8	<0.1	0.5	0.3	1.5	4.1
	dry pomace	5.1	65.1	93.7	40.1	15.2	34.1	4.3	29	27.6	233	309.1
	oil/hexane mixture		151.1	357.3	128.1	54.7	23.1	5.7	149.2	138.4	659.6	1007.6
	crude oil		307.6	576.6	306	117.2	349.8	37.3	465.5	485.3	1540	2645.3
	exhausted pomace	0.2	355	798.3	222.8	81.9	200.5	26	164.9	207.8	1576.6	2057.2
	hexane pre-extraction		<0.1	<0.7	<0.2	<0.1	<0.1	<0.1	<0.3	<0.1	-	-
	hexane post-extraction		<0.1	1.2	0.6	0.4	0.2	0.1	0.6	0.3	2	3.4

## Data Availability

Data is contained within the article.

## Supplementary Materials

The following supporting information can be downloaded at: https://www.mdpi.com/article/10.3390/foods11182737/s1, Figure S1: (A) Operation of moving pomace with bulldozer in the yard of a pomace factory; (B) detail of the bulldozer used to handle the pomace.

## Abbreviations

The following abbreviations are used in this manuscript:

BaA	Benzo(a)anthracene
BaP	Benzo(a)pyrene
BbF	Benzo(b)fluoranthene
BghiP	Benzo(g,h,i)perylene
BkF	Benzo(k)fluoranthene
Ch	Chrysene
DAD	Diode-Array Detection
DBahA	Dibenzo(a,h)anthracene
EFSA	European Food Safety Authority
EU	European Union
FLD	Fluorometric Detector
GC	Gas Chromatography
GC×GC	Comprehensive Two-Dimensional Gas Chromatography
GC-MS	Gas Chromatography–Mass Spectrometry
HPLC	High Performance Liquid Chromatography
IP	Indeno(1,2,3-cd)pyrene
LC	Liquid Chromatography
LC-MS	Liquid Chromatography–Mass Spectrometry
LOD	Limit of Detection
LOQ	Limit of Quantification
MS	Mass Spectrometry
MS-MS	Tandem Mass Spectrometry
OM	Olive Mill
PAH4	BaP, Ch, BaA, BbF
PAH8	BaA, Chr, BbF, BaP, BkF, DBahA, BghiP, IP
PAHs	Polycyclic Aromatic Hydrocarbons
PF	Pomace Factory
RSD	Relative Standard Deviation
SD	Standard Deviation
SPE	Solid Phase Extraction
UV-Vis	Ultraviolet–Visible
