# Supplementary material for: Monitoring and Occurrence of Heavy PAHs in Pomace Oil Supply Chain Using a Double-Step Solid-Phase Purification and HPLC-FLD Determination
Source: Foods. 2022 Sep 6;11(18):2737. doi: 10.3390/foods11182737 (PMC9498164; doi:10.3390/foods11182737)
Supplement: Supplementary file 1 [file foods-11-02737-s001.zip › foods-1846566-supplementary.pdf]

foods

Supplementary Material

Monitoring and Occurrence of Heavy PAHs in Pomace Oil Supply Chain Using a Double-Step Solid-Phase Purification and HPLC-FLD Determination

Laura Barp [1,*], Sabrina Moret [1] and Giorgia Purcaro [2,*]

[1] Department of Agri-Food, Environmental and Animal Sciences, University of Udine, 33100 Udine, Italy
[2] Gembloux Agro-Bio Tech, University of Liège, Chimie des Agro-Biosystèmes, Passage des Déportés 2, 5030 Gembloux, Belgium
* Correspondence: laura.barp@uniud.it (L.B.); gpurcaro@uliege.be (G.P.); Tel.: +32-081-622220 (G.P.)

Abstract: Polycyclic aromatic hydrocarbons (PAHs) are ubiquitous environmental and processing contaminants generated by both spontaneous and anthropogenic incomplete combustion processes of organic matter. Contamination of PAHs in vegetable oils can result from several factors and processes, including environmental contamination, oil processing, and migration from food contact materials. The determination of PAHs in edible oil presents a challenge because of the complexity of the matrix. Since PAHs are present at lower levels than triglycerides, it is necessary to isolate the compounds of interest from the rest of the matrix. To this purpose, a new purification approach based on a double solid-phase extraction (SPE) step followed by high performance liquid chromatography–fluorometric detector (HPLC-FLD) analysis was developed. The method involves a first purification step by using a 5 g silica SPE cartridge, previously washed with dichloromethane (20 mL), dried completely, and then conditioned with *n*-hexane (20 mL). The triglycerides are retained by the silica, while the PAH-containing fraction is eluted with a mixture of *n*-hexane/dichloromethane (70/30, *v*/*v*). After evaporation, the residue is loaded on a 5 g amino SPE cartridge and eluted with *n*-hexane/toluene (70/30, *v*/*v*) before HPLC-FLD analysis. The focus was the evaluation of the contribution of the various phases of the pomace oil supply chain in terms of the heavy PAHs (PAH8) concentration. Data collected showed that pomace contamination increased (by 15 times) as storage time increased. In addition, the process of pomace drying, which is necessary to reduce its moisture content before solvent extraction of the residual oil, appeared to significantly contribute to the total heavy PAHs content, with increases in value by up to 75 times.

Keywords: polycyclic aromatic hydrocarbons (PAHs); olive oil; pomace oil; solid-phase extraction (SPE); high performance liquid chromatography-fluorometric detector (HPLC-FLD)

Citation: Barp, L.; Moret, S.; Purcaro, G. Monitoring and Occurrence of Heavy PAHs in Pomace Oil Supply Chain Using a Double Step Solid-Phase Purification and HPLC-FLD Determination. *Foods* **2022**, *11*, 2737. https://doi.org/10.3390/foods11182737

Academic Editors: Thierry Noguer and Tanja Cirkovic Velickovic

Received: 18 July 2022
Accepted: 1 September 2022
Published: 6 September 2022

Publisher's Note: MDPI stays neutral with regard to jurisdictional claims in published maps and institutional affiliations.

Copyright: © 2022 by the authors. Licensee MDPI, Basel, Switzerland. This article is an open access article distributed under the terms and conditions of the Creative Commons Attribution (CC BY) license (https://creativecommons.org/licenses/by/4.0/).

1. Supplementary Material

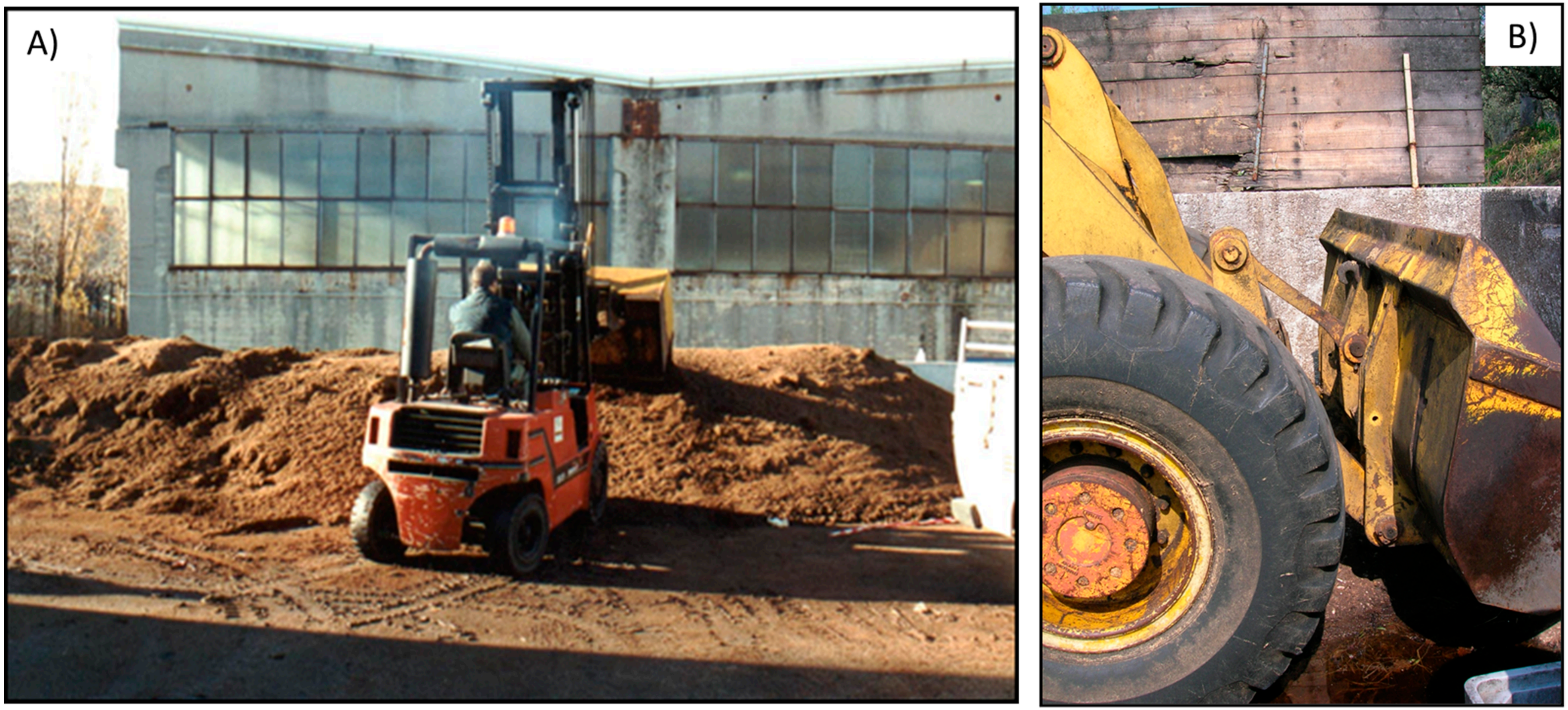

Supplementary Figure S1. (**A**) Operation of moving pomace with bulldozer in the yard of a pomace factory; (**B**): Detail of the bulldozer used to handle the pomace.
